# Ukraine’s EU Integration: A Long Way Home

**DOI:** 10.1007/s10272-022-1066-1

**Published:** 2022-08-06

**Authors:** Ilona Sologoub

**Affiliations:** VoxUkraine, 146 Zhylyanska street, 02000 Kyiv, Ukraine

**Keywords:** F15, F51, F55

## Abstract

It is time to admit that while Russia remains an empire and has nuclear weapons, it will always be an existential threat to democracies.

Ukraine's integration into the EU has many powerful advocates from the European Commission President to about 90% of Ukrainian people (Rating Group Ukraine, 2022). Certainly the process will take some time (hopefully years rather than decades) and will require the transformation of both Ukraine and the EU. However, today the entire world is changing and there will be no return to the reality before 24 February 2022. This new reality requires strategic thinking and bold imagination. The current full-scale war makes it necessary to critically reconsider many things that were perceived as given and finally solve many problems that have been shelved for a long time.

This paper does not try to provide a comprehensive overview of the implementation of the EU-Ukraine Association Agreement – there are quite a few excellent studies that do this (see e.g. Emerson et al., 2021; Ukraine-Europe, 2021). Rather, it discusses several aspects of EU-Ukraine relations and highlights questions that will need to be answered together by Ukraine and the EU when they undergo this journey to accession.

## Political relations

The EU invited Ukraine to the dialogue on 2 December 1991, the day after the Ukrainian people expressed their wish to live in an independent state at a referendum. This event can be called the start of Ukraine-EU relations. They have never been simple (see Table 1). There were both "springs" and "winters" but at the decisive moments, the Ukrainian people defended their democracy and their right to be in the EU. Until now, Ukrainians have been better at uniting against an enemy than around the implementation of reforms. However, after a few centuries of Russian oppression, Ukrainian political culture is gradually developing. On the other hand, looking at the history of continuous attempts to erase Ukrainian language, culture, memory and millions of Ukrainian people, it is a miracle that Ukraine is still alive and fighting. This means that the Ukrainian idea is very resilient. At the same time, this idea is very simple – to have a "normal" nation state, similar to Poland or Lithuania and to eventually rejoin the European family, which Ukraine has been a part of for most of its history.

Since 1991 Ukraine has gone a long way from an autocracy with a planned economy, where entrepreneurship was prosecuted and prices were set by the state, to a market-based democracy, however imperfect. With the help of the International Monetary Fund (IMF), the EU, other governments and international organisations, Ukraine has implemented many reforms, especially since 2014. Certainly, its progress could have been faster. Unfortunately, the legacy of Russian oppression has been very strong. However, over the past 20 years the idea of European integration has spread from a group of enthusiastic technocrats to nearly the entire society.

Since 2014, the majority of Ukrainians support European integration. EU membership will become an anchor for the post-war reconstruction of Ukraine, which will require not only physical reconstruction but also modernisation of institutions. Ukraine has strong economic and personal ties with Europe (see Figures 1, 2 and 3), and given that five million refugees are now hosted in the EU, these ties will become even stronger.

## Economic relations

The EU has always been one of the main trading partners of Ukraine, and since 2014 it is the main trading partner (Figures 1 and 2). After Russia attacked Ukraine in 2014, the EU became the main destination for Ukrainian labour migrants: In 2014-2019 the EU issued 2.8 million permits to Ukrainians for remunerated activities (Dubenko and Kravchuk, 2021).

The EU accounts for about 70% of foreign direct investment (FDI) in Ukraine (although this is partially Ukrainian money previously transferred to Cyprus or other offshores, see Figure 3). And, according to the National Bank of Ukraine data, over 90% of FDI from Ukraine goes to the EU.

The Deep and Comprehensive Free Trade Area (DCFTA) opened new opportunities for Ukrainian businesses – in 2020, 40% of them reported that the EU integration was beneficial for them, about 6% felt worse off, and the rest

Open Access funding provided by ZBW – Leibniz Information Centre for Economics.

**Ilona Sologoub**, VoxUkraine, Kyiv, Ukraine.

Table 1
**Milestones of Ukraine-EU cooperation**

| Date | Milestone |
|---|---|
| 2 December 1991 | In the Declaration on Ukraine, the European Union noted the democratic character of the All-Ukrainian Referendum and called on Ukraine to maintain an open and constructive dialogue with the EU. |
| October 1993 | Kyiv opening of the European Commission Representation in Ukraine. |
| 14 June 1994 | A Partnership and Cooperation Agreement between Ukraine and the EU is signed. |
| 1 June 1995 | Temporary Agreement on Trade and Issues Related to Trade Between Ukraine and the European Community, the European Coal and Steel Community and the European Atomic Energy Community is signed. |
| July 1995 | The Mission of Ukraine to the European Union is established. |
| June 1996 | The European Union recognised the status of Ukraine as a country with a transitional economy. |
| 1 March 1998 | Partnership and Cooperation Agreement between Ukraine and the EU came into force. |
| 11 June 1998 | The Decree of the President of Ukraine approved the Strategy of Ukraine's integration to the EU. |
| 10 December 1999 | The European Council approved the EU Common Strategy on Ukraine aimed at strengthening the strategic partnership between Ukraine and the EU. |
| 11 October 2000 | The resolution of EU Council removing Ukraine from the list of non-market economies became effective. |
| 15 March 2001 | The European Parliament adopted a Resolution on the EU Common Strategy on Ukraine. |
| February 2005 | A Joint EU-Ukraine Action Plan (a framework for key reforms in Ukraine) was endorsed by the European Council. |
| March 2007 | EU and Ukraine started talks about a new "wider agreement", aiming at offering a legal framework for a closer economic cooperation, including a free trade area, and a better political dialogue. |
| 18 February 2008 | Talks on free trade agreement between Ukraine and EU started. |
| 29 October 2008 | Negotiations on visa-free travel started. |
| 2009 | Eastern Partnership cooperation mechanism established for Ukraine and five other post-Soviet countries. |
| 30 March 2012 | The EU-Ukraine Association Agreement (AA) was initiated. |
| 2012 | Ukraine-EU relations deteriorated because then-president Yanukovych jailed the opposition leaders (Yuriy Lutsenko and Yulia Tymoshenko). Later he released Lutsenko. |
| 29 November 2013 | Yanukovych refuses to sign the AA at the Vilnius summit at the insistence of Russia. Euromaidan protests begin. |
| 21 March 2014 | Political part of the Association Agreement signed by the Prime Minister Yatseniuk. |
| 27 June 2014 | Economic part of the AA signed by the President Poroshenko. |
| 16 September 2014 | AA ratified by Ukraine. |
| December 2014 | The EU Advisory Mission for Civilian Security Sector Reform Ukraine is deployed. |
| 1 January 2016 | The Deep and Comprehensive Free Trade Area between Ukraine and the EU entered into force. |
| April 2016 | Referendum in Netherlands on AA ratification (the result is a "no"). Other EU states have ratified the AA by that time. |
| December 2016 | To address the results of the referendum, EU member state governments decided to make legally binding clauses of the AA that stated that the EU did not commit to grant Ukraine EU membership candidate status, provide security guarantees, military or financial aid, or free movement within the EU. |
| 11 May 2017 | Ukraine was granted visa-free travel with the EU. |
| 1 September 2017 | AA fully enters into force. |
| 2021 | An annual dialogue between EU and Ukraine on cybersecurity and cyber defense is launched. |
| 12 October 2021 | Ukraine and the EU sign the Common Aviation Area Agreement, and agreements on Ukraine's participation in the EU Horizon Europe and Creative Europe programmes. |
| 17 December 2021 | The National Energy and Utilities Regulatory Commission of Ukraine certified Ukrenergo as a European-type transmission system operator according to the ISO model. This opened the door to official membership in ENTSO-E. |
| 28 February 2022 | Ukraine submitted an application to join the EU. |
| April 2022 | Ukraine filled in the questionnaire for consideration by the EU Commission. |
| 26 April 2022 | Ukraine became an observer member of ENTSO-E. In March, Ukrainian grid was synchronised with the EU one and disconnected from Russia and Belarus. |
| June 2022 | Ukraine is granted EU candidate status. |

Sources: Compiled by author based on the data of Wolczuk (2003), Ministry of Foreign Affairs of Ukraine, and Ukraine-Europe (2021).

Figure 1
**Ukraine-EU trade in goods**

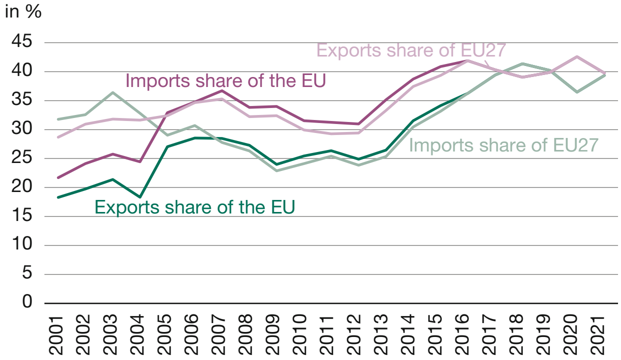

Source: State Statistics Service of Ukraine.

Figure 2
**Ukraine-EU trade in services**

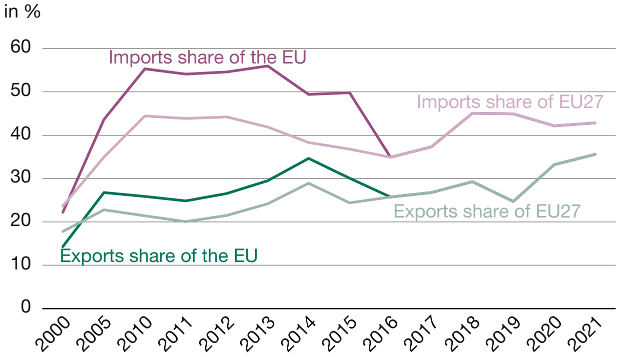

Source: State Statistics Service of Ukraine.

Figure 3
**EU share of foreign direct investment to Ukraine**

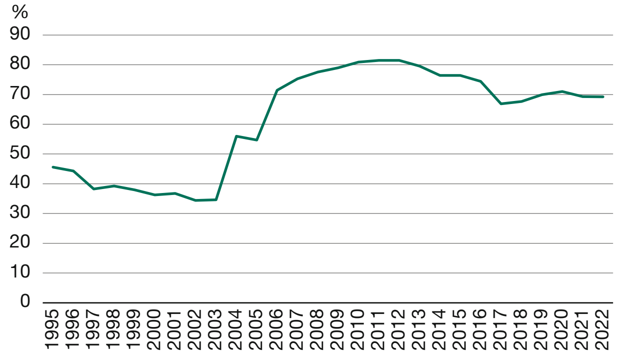

Source: State Statistics Service of Ukraine and National Bank of Ukraine.

did not feel any changes (European Pravda, 2020). As it is unlikely that Ukraine will renew economic ties with Russia any time soon, the importance of the European market for Ukraine will increase, and Ukraine will become more economically and logistically integrated with the EU.

## Popular perception

Looking at the past period since 1991, we can say that until recently Ukraine's progress was driven by a motivated minority. Indeed, in 1991 communists held a majority in the first democratically elected parliament of Ukraine. Despite this, the national democrats, backed by thousands of people in the streets, managed to persuade communists to vote for Ukraine's independence, which was later supported by the majority of Ukrainians in a referendum (84% participated in the referendum and over 90% said "yes" to independence (Commission on Security and Co-operation in Europe, 1992)).

In the early 2000s, European integration was promoted by a few people within the government who were concentrated in the Ministry of Foreign Affairs and the Ministry of Economy and European Integration (Wolczuk, 2003) while president Kuchma was pursuing his "multi-vector" policy. Nevertheless, at that time an important work on the harmonisation of Ukraine's legislation with EU laws was implemented.

An Ilko Kucheriv Democratic Initiatives Foundation (DIF) survey performed in September 2004 showed that 49% of Ukrainians believed that Ukraine would be better off in a union with Russia and Belarus while 29% believed that it would be better off in the EU (DIF, 2004). Yet, two months later Ukrainians came to the streets to protect their electoral choice and democracy. In 2007-08, public opinion moved towards the EU: Polls taken at the time show that the majority of those who would participate in a referendum on joining the EU would vote in favour (DIF, 2008). However, when a survey question included a choice between the EU and Russia, we can see that until 2014 many Ukrainians believed that it was possible to integrate in *both* directions (Figure 4).[1] Other surveys corroborate this result. For example, the IRI (Rating Group Ukraine, 2019) and KIIS surveys (Petrenko, 2016) show that in 2012-13 the shares of Ukrainians who favoured joining the EU and the Customs Union led by Russia were roughly equal. But supporters of EU integration (as well as Putin) understood that the signing of the Association Agreement would be the "point of no return" for Ukraine (Spiliopoulos, 2014). More importantly, they were ready to actively protect their interests.

About 20% of Ukraine's population participated in Euromaidan in all regions of Ukraine (DIF, 2014). This is a

1 This opinion seems strange today but one may remember that for quite a long time the EU was pursuing a "Russia first" policy, and some of its politicians even talked about "Europe from Lisbon to Vladivostok".

Figure 4

**Answers to the question "What is your attitude towards joining the EU or the union with Russia and Belarus"?**

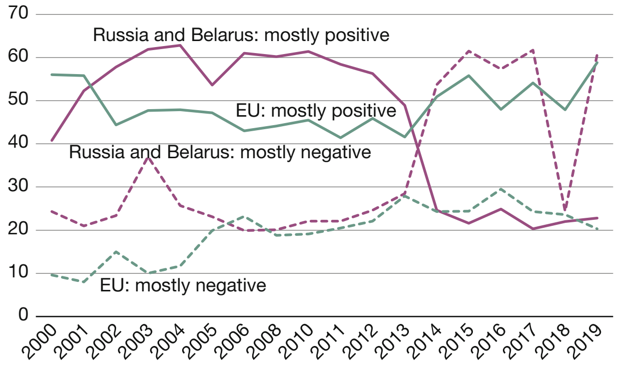

Source: Social monitoring surveys of the Institute of Sociology of the National Academy of Sciences of Ukraine.

minority but it changed the course of the country. Since the Euromaidan, a clear majority of Ukrainians have been supporting EU integration (Figure 4). Perhaps some of these people were "convinced" by the Russian attack on Ukraine in 2014. A recent survey suggests that Russia became even more "convincing" – the share of supporters of EU integration increased from 55%-65% in 2016-2020 to 91% in March 2022 (Rating Group Ukraine, 2022).

What about the Europeans? Are they ready to welcome Ukrainians in the EU? Recent surveys show that between 66% and 71% of Europeans support Ukraine's admission (Eurobarometer, 2022; Finchelstein et al., 2022).

## Ukraine's reforms

The EU, along with the IMF, the World Bank, other governments and international organisations, have been promoting the reforms in Ukraine since the early 1990s – first under the Technical Assistance to the Commonwealth of Independent States programme, later under Twinning and other arrangements. Within the macrofinancial assistance programme, the EU disbursed nearly €6 billion to Ukraine since 2014. Since the start of the full-scale attack on Ukraine, the EU provided Ukraine €1.2 billion under this programme and promised to secure an additional loan of €9 billion in 2022 (European Commission, 2022a).

After 2014 the reforms have considerably intensified. The major factors behind this were the signing of the Association Agreement (AA) with the EU, the existential threat for the country, increased civic activism and "money in exchange for reforms" programmes implemented by the IMF and the EU. Latest studies show that Ukraine was rather successful in implementing the AA. Emerson et al. (2021) suggest that of 26 AA Chapters, Ukraine implemented 17 at a score 2 or higher (on a scale from 1 to 3). The most problematic areas in their view are anti-corruption, rule of law and transport, while civil society received the highest score.

The Ukrainian government estimates that as of 2021, Ukraine implemented 63% of the AA clauses with the highest progress in political dialogue, humanitarian policy as well as justice, freedom and security and human rights protection, while financial cooperation, labour relations and transport lag behind (Ukraine-Europe, 2021).

As Lough et al. (2017) note, the Association Agreement and DCFTA were designed to bring Ukraine closer to the EU (without promising full membership), and some of the clauses were overly complicated given the state of institutional development of Ukraine. Nevertheless, the progress of reforms since 2014 has been substantial. According to VoxUkraine estimates,[2] between January 2015 and June 2022, almost 1,300 reformist legislative acts have been adopted with the most progress in business environment and governance. Of these legislative acts, 127 tackled corruption, 59 indirectly, i.e. by changing the procedures (the most prominent example is the public procurement reform), opening data or deregulating certain spheres. As a result, Ukraine's corruption perception score improved from 25 in 2013 to 32 in 2021; for comparison, Hungary's score fell from 54 to 43 over the same period (Transparency International, 2021).

Since 2014, Ukraine has shown a lot of improvement not only in public attitude to corruption (the share of people who gave bribes declined, while the share of people who cannot justify corruption under any circumstances grew – see Gorodnichenko et al., 2022) but also in the establishment of formal institutions that fight corruption. Thus, National Anti-Corruption Bureau (2021) reports about 859 active investigations in the second half of 2021, and the Higher Anti-Corruption Court completed hearings on more than 110 cases since its launch in 2019; 58 people were convicted.[3] There were attempts by the establishment to reverse some of the anti-corruption developments but the active civil society position helped to reverse those attempts (Euronews, 2020). Certainly, there are remaining problems, of which the unfinished judicial reform is the most important. This reform, as well as anti-corruption reform, is high on the popular agenda. For example, a DIF (2019) survey shows that the five most im-

2 See http://imorevox.org/releases-pdf/.
3 See https://hcac.court.gov.ua/hcac/gromadyanam/analysis/.

portant reforms for Ukrainian citizens are anti-corruption (63%), healthcare (57%), pension and social security reform (52%), reform of judiciary and prosecution (37%) and lustration (33%). Certainly, today Ukrainians care most about winning the war. For if there is no Ukraine then the level of corruption would not matter. However, during the reconstruction, which hopefully will be led by the EU, the interests of Ukrainians and the European institutions will be very much aligned (European Commission, 2022b).

Despite these problems, Ukraine is as qualified for candidate status as the Western Balkan states (Emerson et al, 2022). Provision of the candidate status has no downsides since this status does not foresee any specific admission dates. At the same time it has a huge upside: It gives moral support to the Ukrainian people during the war and, more importantly, provides an anchor for further reforms (an additional bonus is proving Putin, who said that Ukraine would never become an EU member (VoxUkraine, 2021), wrong). Ukraine's path for reforms is rather clear and has been described, for example, in the IMF programmes, European Commission (2020) recommendations or papers on Ukraine reconstruction (Becker et al., 2022). As already mentioned, the most important is completion of the judicial reform, followed by reform of the public service (the decision-making in the public sphere) and reforms that develop markets, including antitrust. Continuing decentralisation is also very important – this is one of the most successful and most popular reforms.

Ukraine's admission to the EU would be beneficial not only for Ukraine but also for the EU itself. One obvious benefit is security: If Ukraine was not currently fighting, Russian tanks would probably already be in Warsaw or Tallinn. In peaceful times, there are many opportunities for cooperation. Obvious spheres are food security and energy production; besides, Ukraine has a lot of human capital and entrepreneurial talent, and it is quite advanced in IT, machine building and other industries that require high-level technical skills. Ukrainian culture is rich and authentic.

However, during the admission process not only Ukraine will change. The EU itself will reform in response to the new challenges. And it will need to answer a few important questions.

### How to modernise the EU?

The necessity of reforms strengthening European unity has been discussed for quite a while. This discussion includes several issues. First, a mechanism of decision-making other than unanimity (Morcos, 2022). As the situation with the sixth package of sanctions showed, Russia can find a "weak link" in the EU and effectively block its decision or cause discord. Second, common or much more aligned fiscal policy (Sapir, 2022). The latest debt crisis in Greece required a lot of money and effort for the sake of saving the eurozone (Gorodnichenko and Korenok, 2015). Third, common foreign policy, a part of which is further EU enlargement, e.g. there is a proposal on staged accession to the EU in order not to discourage Balkan states (Emerson and Blockmans, 2022). If adopted, this procedure can be also applied to Ukraine, Georgia and Moldova.

At the same time, adoption of the EU regulations by candidate states may revitalise the debate on the review of European regulations. Deregulation would make the EU more competitive compared to the US or China.

In short, a larger EU requires more efficient decision-making mechanisms. At the same time, the EU that speaks with one voice can become a much stronger international player. Since the EU is based on values such as respect for human rights, freedom and democracy, this will help to make the world a more democratic and safer place (democracies are less likely to unleash wars, see e.g. Mintz and Geva (1993)). This has direct implications for regional and global security. Recall that the EU was based on the very simple idea of preventing another war in Europe by making European countries as economically intertwined as possible. This did not work with Russia because it is not a democracy. Thus, it is time to rethink the basic idea of the EU and at the same time answer other important questions.

### What to do with Russia?

The *realpolitik* idea rooted in the mid-20th century proved to be wrong. Turning a blind eye to violations of human rights and international laws did not pacify Russia (nor will they pacify China or other autocracies).

The European Council (2022) in its recent statement seems to realise this. At least it demands that Russia withdraw its troops from the entire territory of Ukraine and recognises the need to reduce the EU strategic dependence on Russia.

However, this is not enough. It is time to admit that Russia's values are the opposite of EU basic values. There is no respect for human rights in Russia, no freedom or democracy and no rule of law. Moreover, Russia, as well as the USSR before 1991, tries to undermine these values whenever it can. In fact, today's Russia is nothing new. It is the same as Germany in the 1930s or the USSR throughout its history (Marayev and Guz, 2022). Its external and intenal policy is terror supported by a large part of the population (Levada Center, 2022; Zholud and Sologoub, 2022).[4] Therefore, it should be recognised as a terrorist state and treated accordingly.

To become a "normal" nation, the Russian imperial project should be defeated in the same way as Nazi Germany and Imperial Japan. And this is not only a Ukrainian endeavor. The outcome of this war will have long-lasting implications for both the EU and the world. While economic implications of the war for the EU seem rather modest (They are smaller than the consequences of the COVID-19 pandemic in terms of GDP decline, see Blanchard and Pisani-Ferry (2022)), its political and security consequences will be huge. Russia's threat to the Eastern European states as well as to Central Asian countries such as Kazakhstan is very real. Other countries, first of all China, are now discovering the ability of the collective West to protect its values. Thus, any scenario that involves further "appeasement" of Russia will be a threat not only to millions of Ukrainians. This scenario will enable multiple armed conflicts around the world (many of them will be spurred by Russia).

It is time to admit that while Russia remains an empire and has nuclear weapons, it will always be an existential threat to democracies. Thus, the EU should start communications with the civil society and possible leaders of the new independent states that will emerge after the demise of Russia (the obvious candidates are Ichkeria (Chechnya) that fought for its independence for over a decade (Roland, 2022), as well as Karelia, Tatarstan, Komi and Yakut Republics that declared their independence in 1989-90 (Corbet and Gummich, 1990)). As the example of Ukraine, Belarus and Kazakhstan shows, it is much easier to convince nation states to give up their nuclear weapons compared to the state that considers itself a "superpower" (Gorodnichenko and Sologoub, 2022). Generally, the "superpower" concept is outdated. If we believe that modern states are based on principles of equal rights and rule of law, these principles should apply not only to people but also to nations. How can we ensure this?

## How to reform the world security system?

The reform of the UN has also been discussed for quite a while. Many countries are upset by the veto power and by the fact that some countries have more rights than others. Many observers are outraged by Russia's conduct and impunity as a permanent security council member. If today's war is not a sufficient incentive to finally start the UN reform, then what is?

Stating the obvious, rules are useless if they are not enforced. Thus, there should be a mechanism that immediately punishes the aggressor state if it attacks another country. If such a mechanism was in place in 2014, then asset freezes and oil embargos as well as a ban on imports would have been automatically applied to Russia as soon as it annexed Crimea. When such a mechanism is created, it would increase the cost of war for authoritarian states (since these are more likely to attack other countries (see Coleman, 2004)) and limit their ability to wage a war.

The world is becoming a more complicated place. A place where the role of natural resources[5] is fading and the role of human capital is rising. Since human capital can be utilised to the full extent only in an environment of personal freedoms and protected human rights, logically the states that provide this environment will win the battle for the future. However, as the war of Russia on Ukraine shows, sometimes nations not only prefer to stay in the past but also try to prevent modernisation of others.

## Conclusions

The current ongoing war is the war for the future. Thus, Ukraine must win. This victory will benefit Europe, the entire world and even (paradoxically) Russia. But today Ukraine urgently needs weapons to reduce the human cost of this victory.
